# Racial disparities in the utilization of preventive health services among older women with early‐stage endometrial cancer enrolled in Medicare

**DOI:** 10.1002/cam4.1141

**Published:** 2017-08-04

**Authors:** Jovana Y. Martin, Melissa A. Schiff, Noel S. Weiss, Renata R. Urban

**Affiliations:** ^1^ Department of Obstetrics and Gynecology University of Washington Seattle Washington; ^2^ Division of Gynecologic Oncology University of Washington Seattle Washington; ^3^ Department of Epidemiology University of Washington School of Public Health Seattle Washington

**Keywords:** Cholesterol screening, diabetes mellitus screening, endometrial neoplasms, healthcare disparities, influenza vaccines, mammography, preventive health services, survivors

## Abstract

To assess differences in the receipt of preventive health services by race/ethnicity among older women with endometrial cancer enrolled in Medicare, we conducted a retrospective population‐based cohort study of women diagnosed with endometrial cancer from 2001 to 2011 in the Surveillance Epidemiology and End Results (SEER)‐Medicare database. Women with stage I or II endometrial cancer of epithelial origin were included. The exposure was race/ethnicity (Non‐Hispanic [NH] White, NH Black, Hispanic, and NH Asian/Pacific Islander [PI]). The services examined were receipt of influenza vaccination and screening tests for diabetes mellitus, hyperlipidemia, and breast cancer. We used multivariate logistic regression to estimate odds ratios with 95% confidence intervals (CI) adjusted for age, region, and year of diagnosis. A total of 13,054 women were included. In the 2 years after diagnosis, receipt of any influenza vaccine ranged from 45% among NH Black women to 67% among NH White women; receipt of a mammogram ranged from 65% among NH Black women to 74% among NH White women. Relative to NH White women, NH Black women had a lower likelihood of receiving both influenza vaccination (adjusted odds ratio [aOR] 0.40, 95% CI 0.33–0.44) and screening mammography (aOR 0.64, 95% CI 0.52–0.79). Hispanic women also were less likely to receive influenza vaccination than NH White women (aOR 0.61, 95% CI 0.51–0.72). There were no significant differences across racial groups for diabetes or cholesterol screening services. Among older women with early‐stage endometrial cancer, racial disparities exist in the utilization of some preventive services.

## Introduction

Endometrial cancer is the most common gynecological malignancy in the US, with an anticipated 60,050 incident cases and 10,470 deaths in 2016 1. The majority of women with endometrial cancer are diagnosed at an early stage and have a favorable cancer‐related prognosis 1. Women with low‐grade endometrial cancer have a high prevalence of concurrent comorbid conditions and are more likely to die from cardiovascular disease than endometrial cancer 2, 3. Given this, preventive medicine and promoting treatment of comorbidities among endometrial cancer survivors is likely to be of substantial benefit.

Preventive health care utilization among cancer survivors has been examined and the quantity of services received has varied 4, 5, 6, 7, 8, 9, 10, 11, 12, 13. At the age of 65, all United States citizens and permanent legal residents are eligible to receive Medicare benefits, eliminating the lack of insurance as a barrier to health care access. Despite this, racial disparities in the utilization of preventive health services exist among Medicare beneficiaries 14. Compared to Whites, Blacks and Hispanics are less likely to receive mammography, colonoscopy, and influenza vaccination 15, 16, 17, 18, 19, 20.

The utilization of preventive services by women with early‐stage endometrial cancer after their cancer diagnosis has not been well described. Given that women with stage I and II endometrial cancer have a favorable overall cancer‐related survival, with 83% of women expected to be alive in 5 years 1, our objective was to assess differences in the receipt of preventive care among Medicare beneficiaries with early‐stage endometrial cancer by race and ethnicity in the survivorship period.

## Methods

We conducted a retrospective population‐based cohort study of women diagnosed with endometrial cancer between 2001 and 2011 in the SEER registries linked to the Medicare database to evaluate the association between race/ethnicity and the receipt of preventive services. The cancer registries that participate in the SEER program cover approximately 28% of the US population 21. Each registry collects data on demographics, stage, histology, and treatment 21. The Medicare files include demographic information and billing claims. With each linkage, 93% of individuals aged 65 years and older in the SEER files are successfully matched to the Medicare files 22.

The linked dataset for this analysis included women diagnosed with uterine cancer from 1 January 2001 to 31 December 2011 with analysis of billing claims from 1 January 2000 to 31 December 2013. The University of Washington Human Subjects Division classified this project as exempt from human subjects research. Women were included in the study if they: were diagnosed with stages I through II endometrial carcinoma of epithelial origin, given the promising long‐term cancer‐related survival of such women; were at least 66 years of age at diagnosis, to allow for a minimum of 1 year of Medicare eligibility; had a hysterectomy as part of the definitive management for their endometrial cancer; had Medicare Part A and Part B for at least 1 year prior to their endometrial cancer diagnosis; and had continuous enrollment in both Medicare parts A and B during the study period. Women were excluded if they had a managed care plan in addition to Medicare, in which case there could be billing claims for screening services that would be inaccessible for analysis. They were also excluded if they were missing information on tumor stage at diagnosis, or had a major secondary cancer diagnosis excluding nonmelanoma skin cancer before their endometrial cancer diagnosis. For the analysis within 2 years of endometrial cancer diagnosis, women were excluded if they had a second cancer diagnosis within the 2 years after their endometrial cancer diagnosis. Similarly, for analysis within 5 years of endometrial cancer diagnosis, women were excluded if they had a second cancer within the 5 years after their diagnosis.

Our exposure of interest for receipt of preventive services was patient race/ethnicity, categorized as Non‐Hispanic (NH) White, NH Black, Hispanic, and NH Asian/Pacific Islander (PI). Priority was given to the race variable from the Medicare Enrollment Database and missing values were filled in from the SEER race variables. Furthermore, we used the Hispanic origin variable from SEER to categorize subjects as Hispanic ethnicity, regardless of their race. Women of any other race/ethnicity were excluded from our analysis. To determine the utilization of preventive services, we examined receipt of services through two time points; 2 years and 5 years after diagnosis. Preventive services for up to 2 and 5 years after diagnosis were chosen because during this time‐frame endometrial cancer survivors would be expected to have visits to providers for not only their routine medical care but also for endometrial cancer surveillance 23. The preventive care services examined included well visits, influenza vaccination, breast cancer screening, cholesterol screening in those without a diagnosis of hyperlipidemia 1 year prior to endometrial cancer diagnosis, and diabetes mellitus screening in those without a diagnosis of diabetes mellitus 1 year prior to endometrial cancer diagnosis. These services were selected because they are preventive services that are billed with CPT codes in Medicare billing claims, and therefore can be measured for analysis. To qualify for diabetes mellitus screening, a woman had to have a diagnosis of obesity, hypertension, or hyperlipidemia 1 year prior to endometrial cancer diagnosis. We followed the United States Preventive Services Task Force (USPTSF) recommendations for frequency of screening and the Centers for Disease Control (CDC) recommendations for frequency of vaccination (Appendix S1).

We assessed the Medicare billing claims to identify the receipt of selected prevention services utilized using Current Procedural Terminology (CPT), International Classification of Diseases (ICD‐9) 9th edition, and Healthcare Common Procedure Coding System (HCPCS) codes outlined in Appendix S2. Additionally, for receipt of preventive services that were age‐specific, we accounted for the patient's age at the time of endometrial cancer. For example, breast cancer screening is not recommended over the age of 74. To account for this in our assessment for breast cancer screening, women diagnosed with endometrial cancer who were over the age of 72 were excluded from analysis. Lastly, to minimize measurement bias, we only considered screening mammography ICD‐9 codes as an indicator for breast cancer screening.

We collected information on the following demographic variables: age at diagnosis, marital status, area of residence (urban vs. rural), geographic region of diagnosis, and year of diagnosis. From the 2000 census tract we determined the median household income, the percent below 100% of the poverty level, and the percent with less than a high school education, categorized into quartiles; the lowest quartile (less than 25th percentile), low through middle (25th–50th percentile), middle to high (>50th percentile–75th percentile), and the highest quartile (>75th percentile). We determined the Charlson comorbidity index score (with the Deyo modification for administrative databases 24, 25) from the medical comorbidities located in inpatient and outpatient Medicare claims 1 year prior to cancer diagnosis. Cancer was excluded from the Charlson comorbidity index score. The cancer treatments determined from Medicare billing claims 6 months after diagnosis were adjuvant radiation therapy and adjuvant chemotherapy.

Demographic, clinical, and treatment factors were compared between race/ethnic groups using chi‐squared tests. We used logistic regression to estimate adjusted odds ratios (aOR) with 95% confidence intervals (CI) to determine the association between race/ethnicity and the receipt of each preventive service with a complete case analysis approach. For each preventive service, we modeled the outcome in two ways. First, we modeled the receipt of the service at least once in 2 years or 5 years. Second, we modeled receipt of the service if it was received at the recommended frequency during the 2 year and 5 year follow‐up time period by the USPSTF. Within 2 years of diagnosis, we looked at receipt of two influenza vaccinations within 2 years, and within 5 years of diagnosis, we looked at the receipt of five influenza vaccinations in 5 years and two screening mammography's within 5 years. Beta coefficients (beta) with 95% confidence intervals were estimated with linear regression to determine if there was a linear trend in the utilization of preventive services over time.

We chose to examine the following variables as potential confounders: year of cancer diagnosis, age at the time of diagnosis, SEER region, obesity, and Charlson comorbidity index score using a causal diagram (Appendix S3) 26. Using the causal relationships in the causal diagram, with the assistance of a computer graphical interface DAGitty 27, age at the time of diagnosis, SEER region, and year of diagnosis were determined to be the set of variables for adjustment required to minimize confounding.

## Results

**Figure 1:**
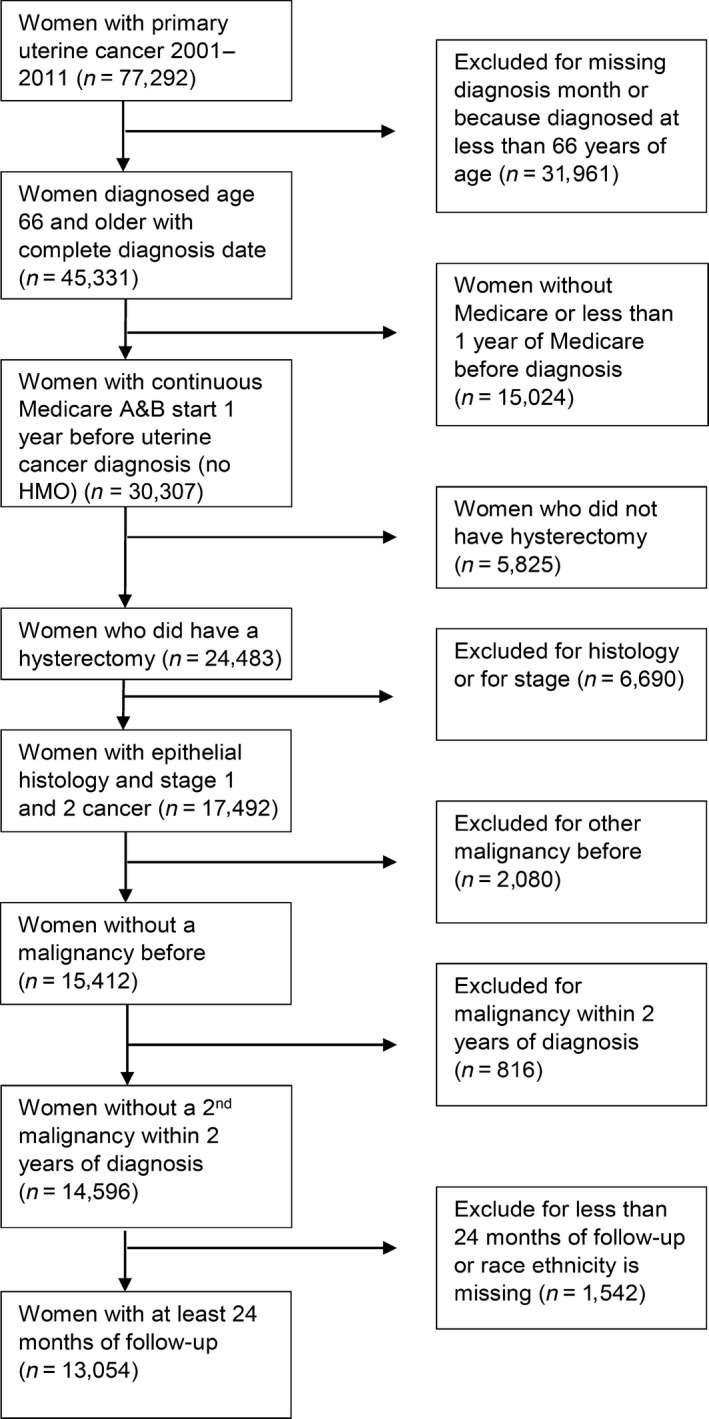
Study flow chart.

From 2001 to 2011, we identified 77,292 women with cancer of the uterine corpus in the SEER‐Medicare database. A total of 13,054 patients were eligible for analysis (Fig. 1). Women with less than 2 years of Medicare follow‐up were more likely to be older, of NH Black race/ethnicity, unmarried, have higher Charlson comorbidity scores, worse tumor grade, and stage II cancers. NH Black, Hispanic, and NH Asian/PI women were more likely to be diagnosed with endometrial cancer at a younger age compared to NH White women (Table 1). NH Black women were less likely to be married and more likely to have stage II cancer and obesity compared to other race/ethnic groups. More NH Blacks, Hispanics, and Asian/PI had diabetes and hypertension compared to NH Whites. Both NH Black and Hispanics were overrepresented in the lowest quartiles for median census tract.

**Table 1:** Characteristics of women diagnosed with endometrial cancer 2001–2011 SEER‐Medicare with at least 2 years of Medicare follow‐up

*n*=13,054	NH White		NH Black		Hispanic		Asian/PI	
	*n*	%	*n*	%	*n*	%	*n*	%
	11,347	86.92	772	5.91	601	4.60	334	2.56
Age at diagnosis								
66–74	6439	56.75	521	67.49	388	64.56	211	63.17
75–79	2417	21.30	151	19.56	118	19.63	88	26.35
80+	2491	21.95	100	12.95	95	15.81	35	10.48
Year of diagnosis								
2001–2004	4614	40.66	273	35.36	235	39.10	107	32.04
2005–2007	3007	26.50	218	28.24	139	23.13	85	25.45
2008–2011	3726	32.84	281	36.40	227	37.77	142	42.51
Marital status								
Married (yes)	5508	48.54	227	29.40	253	42.10	187	55.99
Unknown	390	3.44	27	3.50	18	3.00	9	2.69
Stage at diagnosis								
I	10,218	90.05	653	84.59	533	88.69	302	90.42
II	1129	9.95	119	15.49	68	11.31	32	9.58
Charlson comorbidity score								
0	7739	68.20	388	50.26	347	57.74	197	58.98
1	2426	21.38	237	30.70	172	28.62	91	27.25
>=2	1055	9.30	138	17.88	75	12.48	36	10.78
Obese								
yes	894	7.88	129	16.71	67	11.15	15	4.49
Hypertension^a^								
yes	7730	68.18	647	83.81	443	73.71	249	74.55
Diabetes^a^								
yes	2983	26.29	345	44.69	237	39.43	128	38.32
Grade								
1: Well differentiated	4551	40.11	240	31.09	237	39.43	110	32.93
2: Moderately differentiated	3712	32.71	228	29.53	189	31.45	106	31.74
3: Poorly differentiated	1662	14.65	177	22.93	108	31.45	75	22.46
4: Undifferentiated	284	2.50	45	5.83	18	3.00	18	5.39
Unknown	1138	10.03	82	10.62	49	8.15	25	7.49
Histology								
Endometroid	10,108	89.08	613	79.40	510	84.86	287	85.93
Clear cell	119	1.05	18	2.33	7	1.16	7	2.10
Mucinous	124	1.09	6	0.78	4	0.67	1	0.30
Serous	445	3.92	88	11.40	38	6.32	24	7.19
Other	551	4.86	47	6.09	42	6.99	15	4.49
Radiotherapy								
Yes (%)	3049	26.87	214	27.72	161	26.72	77	23.05
Unknown	9	0.08	0	0.00	1	0.17	0	0.00
Chemotherapy								
Yes (%)	547	4.82	70	9.07	42	6.99	22	6.59
SEER region at diagnosis								
Northeast	2950	26.00	179	23.19	132	21.96	19	5.69
Midwest	1754	15.46	126	16.32	14	2.33	5	1.50
South	2190	19.30	319	41.32	22	3.66	4	1.20
West	4453	39.24	148	19.17	433	72.05	306	91.62
Urban								
Yes (%)	9367	82.55	687	88.99	567	94.34	317	94.91
Median census tract income								
Quartile 1 (lowest)	2501	22.18	465	60.39	214	35.67	60	17.96
Quartile 2 (low‐mid)	2888	25.61	143	18.57	145	24.17	70	20.96
Quartile 3 (high‐mid)	2917	25.87	106	13.77	130	21.67	91	27.57
Quartile 4 (highest)	2971	26.35	56	7.27	111	18.50	113	33.83
Unknown	70	0.62	2	0.26	1	0.17	0	0.00
% below poverty								
Quartile 1 (lowest)	3025	26.82	63	8.18	88	14.67	86	25.75
Quartile 2 (low‐mid)	3015	26.74	74	9.61	82	13.67	71	21.26
Quartile 3 (high‐mid)	2901	25.72	114	14.81	145	24.17	81	24.25
Quartile 4 (highest)	2336	20.71	519	67.40	285	47.50	96	28.74
Unknown	70	0.62	2	0.26	1	0.17	0	0.00
% less than HS education								
Quartile 1 (lowest)	3052	27.06	44	5.71	75	12.50	80	23.95
Quartile 2 (low‐mid)	2991	26.52	92	11.95	93	15.50	68	20.36
Quartile 3 (high‐mid)	2908	25.79	145	18.83	119	19.83	75	22.46
Quartile 4 (highest)	2326	20.63	489	63.51	313	52.17	111	33.23
Unknown	70	0.62	2	0.26	1	0.17	0	0.00
Death								
Yes	2996	26.14	220	28.50	145	24.13	64	19.16

Diagnosed 1 year prior to endometrial cancer.

We found that receipt of any influenza vaccination within 2 years of diagnosis varied by race, with a range of 45% for NH Black women to 67% for NH White (Table 2). We found that relative to NH White women, the odds of receiving any influenza vaccination were 0.40 for NH Black women (95% CI 0.33–0.44) and 0.61 for Hispanic women (95% CI 0.51–0.72). The corresponding odds ratios for receipt of two influenza vaccinations in 2 years were 0.33 (95% CI 0.27–0.39) and 0.47 (95% CI 0.39–0.57) for NH Black women and Hispanic women compared to NH White women, respectively. The proportion of women who received five influenza vaccinations within 5 years ranged from 8% for NH Black women to 26% for NH White women (Table 3). The odds of receiving five influenza vaccinations within 5 years were 0.23 for NH Black women (95% CI 0.16–0.34) and 0.39 for Hispanic women (95% CI 0.27–0.56).

**Table 2:** Preventive services odds ratios by race/ethnicity for women diagnosed with endometrial cancer 2001–2011 SEER‐Medicare with at least 2 years of follow‐up

	NH White		NH Black		Hispanic		Asian/PI	
	*n*=11,347	86.92%	*n*=772	5.91%	*n*=601	4.60%	*n*=334	2.56%
Screening service								
Well visit (*n* = 13,054)								
Yes (%)	1374	12.11	78	10.10	70	11.65	49	14.67
OR (95% CI)	ref	ref	0.82	0.64, 1.04	0.96	0.74, 1.24	1.25	0.92, 1.70
aOR (95% CI)^d^	ref	ref	0.82	0.64, 1.05	0.81	0.62, 1.05	0.98	0.72, 1.35
Influenza vaccination (*n* = 13,054)								
Yes (%)	7643	67.36	347	44.95	317	52.75	222	66.47
OR (95% CI)	ref	ref	0.40	0.34, 0.45	0.54	0.46, 0.64	0.96	0.76, 1.21
aOR (95% CI)^d^	ref	ref	0.40	0.33, 0.44	0.61	0.51, 0.72	1.15	0.91, 1.45
2 Influenza vaccinations a(*n* = 13,054)								
Yes (%)	4828	42.55	155	20.08	143	23.79	136	40.72
OR (95% CI)	ref	ref	0.34	0.28, 0.41	0.42	0.35, 0.51	0.93	0.74, 1.15
aOR (95% CI)^d^	ref	ref	0.33	0.27, 0.39	0.47	0.39, 0.57	1.08	0.86, 1.36
Breast cancer screening^b^ (*n* = 6145)								
Yes (%)	3876	74.15	283	65.36	218	69.21	117	68.82
OR (95% CI)	ref	ref	0.66	0.53, 0.81	0.78	0.61, 1.00	0.77	0.55, 1.07
aOR (95% CI)^d^	ref	ref	0.64	0.52, 0.79	0.82	0.64, 1.05	0.82	0.58, 1.14
Diabetes screening^c^ (*n* = 5933)								
Yes (%)	1684	32.23	123	37.85	94	37.75	54	40.30
OR (95% CI)	ref	ref	1.28	1.01, 1.61	1.28	0.98, 1.66	1.42	1.00, 2.01
aOR (95% CI)^d^	ref	ref	1.34	1.06, 1.71	1.35	1.03, 1.77	1.47	1.02, 2.10

a Restricted to women that had two influenza vaccinations within 2 years of endometrial cancer diagnosis.

b Restricted to women diagnosed age 72 and younger.

c Restricted to those without a diagnosis of diabetes 1 year prior to cancer diagnosis and with history of obesity, hypertension, or hyperlipidemia.

d Adjusted for year of diagnosis, region, and age at diagnosis.

**Table 3:** Preventive services and odds ratios by race/ethnicity for women diagnosed with endometrial cancer 2001–2011 SEER‐Medicare with at least 5 years of follow‐up

	NH White		NH Black		Hispanic		Asian/PI	
	*n* = 6638	88.22%	*n* = 391	5.20%	*n* = 328	4.36%	*n* =167	2.22%
Screening service								
Well visit (*n* = 7524)								
Yes (%)	1403	21.14	82	20.97	65	19.82	30	17.96
OR (95% CI)	ref	ref	0.95	0.74, 1.22	0.95	0.72, 1.22	0.88	0.60, 1.28
aOR (95% CI)^e^	ref	ref	1.04	0.80, 1.34	0.81	0.61, 1.08	0.65	0.43, 0.98
Influenza vaccination (*n* = 7524)								
Yes (%)	5245	79.01	233	59.59	226	68.90	130	77.84
OR (95% CI)	ref	ref	0.39	0.32, 0.48	0.59	0.47, 0.75	0.91	0.64, 1.30
aOR (95% CI)	ref	ref	0.39	0.31, 0.48	0.65	0.51, 0.83	1.09	0.75, 1.59
5 Influenza vaccinations^a^ (*n* = 7524)								
Yes (%)	1758	26.48	32	8.18	36	10.98	37	22.16
OR (95% CI)	ref	ref	0.26	0.18, 0.36	0.34	0.24, 0.48	0.84	0.59, 1.20
aOR (95% CI)	ref	ref	0.23	0.16, 0.34	0.39	0.27, 0.56	0.95	0.65, 1.39
Breast cancer screening^b^ (*n* = 3595)								
Yes (%)	1981	85.28	132	77.65	102	80.95	56	80.00
OR (95% CI)	ref	ref	0.66	0.47, 0.93	0.62	0.43, 0.88	0.82	0.48, 1.40
aOR (95% CI)	ref	ref	0.65	0.49, 0.92	0.61	0.42, 0.88	0.80	0.46, 1.38
At least 2 breast cancer screenings^b^ (*n* = 3595)								
Yes (%)	1697	73.05	102	60.00	90	71.43	49	70.00
OR (95% CI)	ref	ref	0.57	0.43, 0.76	0.85	0.62, 1.18	1.01	0.67, 1.68
aOR (95% CI)	ref	ref	0.56	0.41, 0.75	0.84	0.60, 1.18	1.01	0.63, 1.62
Diabetes screening^c^ (*n* = 3455)								
Yes (%)	1551	50.54	95	57.58	94	61.04	43	64.18
OR (95% CI)	ref	ref	1.44	1.06, 1.97	1.56	1.13, 2.16	1.68	1.03, 2.73
aOR (95% CI)	ref	ref	1.38	1.00, 1.91	1.59	1.14, 2.23	1.85	1.11, 3.09
Cholesterol screening^d^ (*n* = 7524)								
Yes (%)	6058	91.26	361	92.33	300	91.46	154	92.22
OR (95% CI)	ref	ref	1.18	0.80, 1.72	1.02	0.69, 1.50	1.18	0.67, 2.09
aOR (95% CI)	ref	ref	1.14	0.77, 1.68	0.95	0.64, 1.43	1.01	0.56, 1.80

a Restricted to women who had five influenza vaccinations within 5 years of endometrial cancer diagnosis.

Screening mammogram; restricted to women diagnosed age 72 and younger.

b At least two screening mammograms; restricted to women diagnosed age 72 and younger.

c Restricted to those without a diagnosis of diabetes 1 year prior to cancer diagnosis and with hx of obesity, hypertension, or hyperlipidemia.

d Restricted to those without diagnosis of hyperlipidemia 1 year prior to endometrial cancer diagnosis.

e Adjusted for year of diagnosis, region, and age at diagnosis.

Within 2 years of diagnosis, the proportion of women who received at least one mammogram ranged from 65% for NH Black women to 74% for NH White women (Table 2). Compared to NH White women, the odds for receiving any mammogram within 2 years of diagnosis was 0.64 for NH Blacks (95% CI 0.52–0.79). Within 5 years of diagnosis, the odds for receiving any mammogram for Hispanic women was 0.61 (95% CI 0.42–0.88) and 0.65 for NH Black women (95% CI 0.49–0.92) compared to NH White women (Table 3). We examined the frequency of having at least two mammograms within 5 years of diagnosis, and found that the odds were 0.84 for Hispanic women (95% CI 0.60–1.18) and 0.56 for NH Black women (95% CI 0.41–0.75) compared to NH White women (Table 3).

Over 50% of women in each race/ethnicity category received at least one diabetes mellitus screening test within 5 years of diagnosis, with a range of 51% for NH White women to 64% for Asian/PI women (Table 3). Relative to NH White women, Hispanic women (aOR 1.59 95% CI 1.14–2.23), NH Black women (aOR 1.38 95% CI 1.00–1.91), and NH Asian/PI women (aOR 1.85 95% CI 1.11–3.09) had a higher likelihood of receiving at least one diabetes mellitus screening test within 5 years.

Receipt of cholesterol screening was similar between race/ethnicity groups, with 90% of women in each group having at least one cholesterol screening test within 5 years of diagnosis (Table 3).

**Figure 2:**
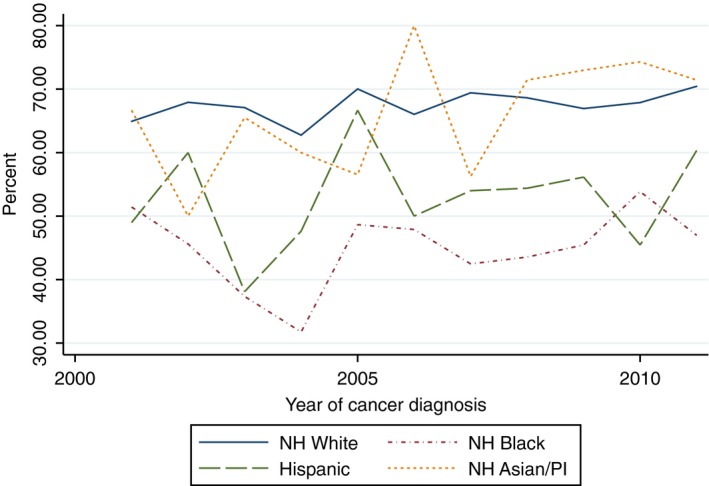
At least one influenza vaccine within 2 years of diagnosis trends over time.

**Figure 3:**
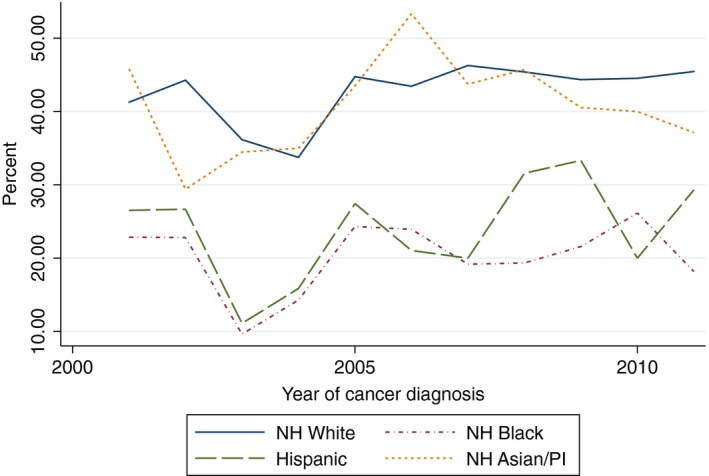
Two influenza vaccines within 2 years trends over time.

**Figure 4:**
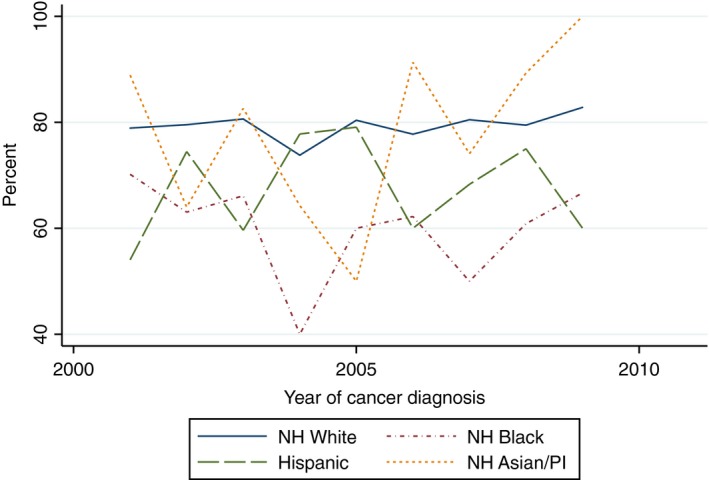
At least one influenza vaccine within 5 years of diagnosis trends over time.

**Figure 5:**
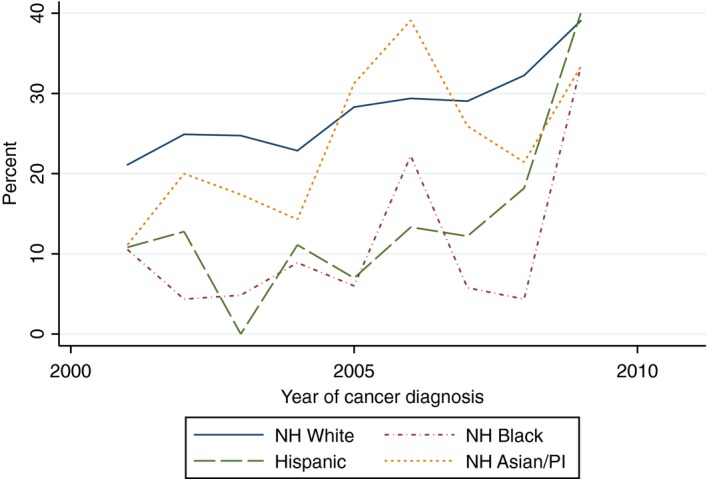
Five influenza vaccines within 5 years trends over time.

**Figure 6:**
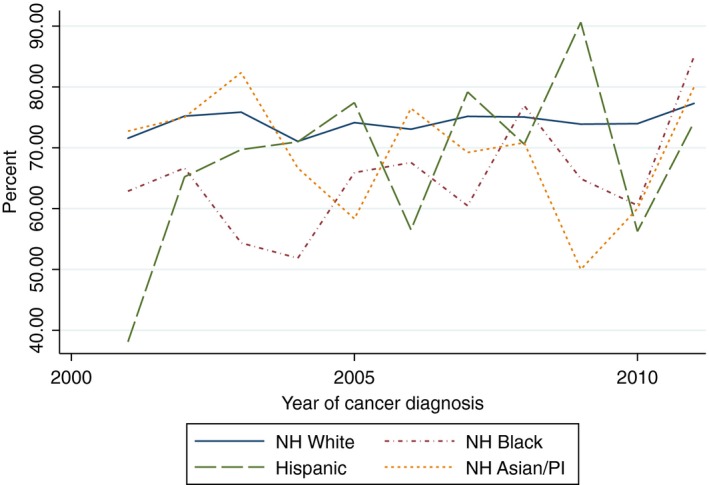
At least one mammogram within 2 years of diagnosis trends over time.

**Figure 7:**
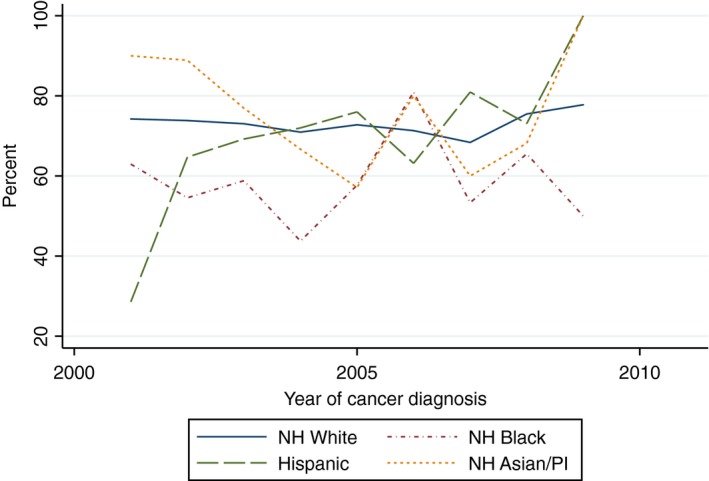
Two mammograms within 5 years trends over time.

**Figure 8:**
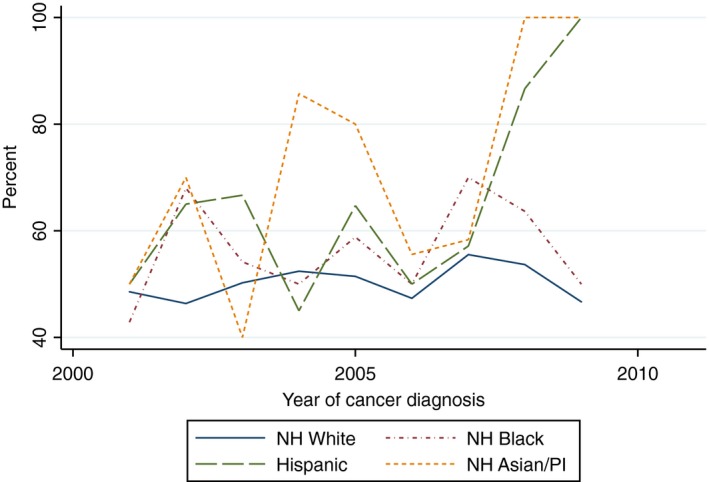
Diabetes screening test within 5 years of diagnosis trends over time.

**Figure 9:**
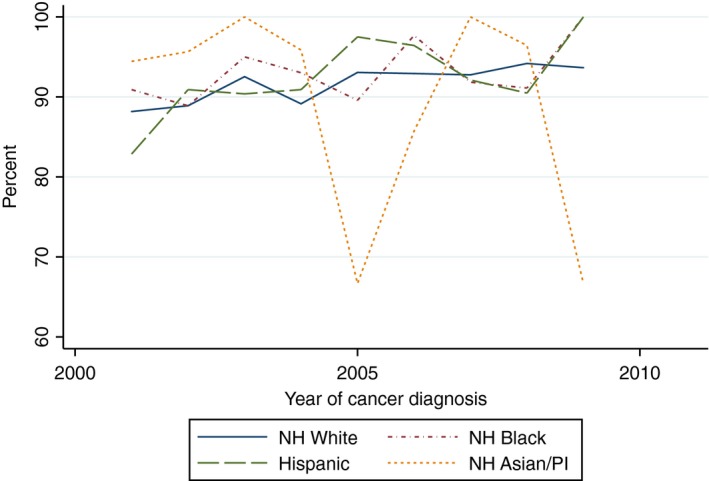
Cholesterol screening within 5 years of diagnosis trends over time.

There was a statistically significant increasing linear trend in the proportion of White women who received yearly influenza vaccinations within the 5 years after cancer diagnosis (Beta 1.82, 95% CI 1.09–2.54) and the proportion of Hispanic women who received two mammograms within 5 years of diagnosis (Beta 5.42, 95% CI 1.60–9.24). With the exception of the aforementioned, there was no evidence of a statistically significant trend in the proportion of women of all race/ethnicities who received influenza vaccinations, mammograms, diabetes mellitus screening, or lipid tests (Figs. 2, 3, 4, 5, 6, 7, 8, 9).

## Discussion

Among women with newly diagnosed early‐stage endometrial cancer who were identified in the SEER‐Medicare database from 2001 to 2011, we found that racial disparities existed in the utilization of influenza vaccinations, and screening mammography. During the first 5 years following an endometrial cancer diagnosis, relative to NH White women, NH Black and Hispanic women had a lower likelihood of receipt of influenza vaccination, and NH Black women had a lower likelihood of receipt of screening mammography. We also found that relative to NH White women, NH Black women, Hispanic women, and NH Asian/PI women had a higher likelihood for receipt of diabetes mellitus screening. The Healthy People 2020 target is 81% for screening mammography, 82% for cholesterol screening, and 90% for influenza vaccination. With the exception of cholesterol screening, women of all racial and ethnic backgrounds with early‐stage endometrial cancer are well below these targets for screening and vaccination. Furthermore, with the exception of influenza vaccination in NH White women and mammograms in Hispanic women within 5 years of endometrial cancer diagnosis, there was no evidence of an increasing trend in the receipt of vaccination or screening services among cancer survivors.

The Healthy People 2020 target for vaccination is 90% 28, and we have shown that early‐stage endometrial cancer survivors in the US are receiving influenza vaccinations well below this target, with a range of 45% for NH Black women to 67% for NH White women. A previous study by McBean et al. looked at the frequency of preventive services among endometrial cancer survivors more than 5 years after endometrial cancer diagnosis, while in our analysis, we looked at the frequency of services from the time of diagnosis until 5 years after in order to gain understanding of the preventive services use of survivors undergoing surveillance for endometrial cancer 8. They found that Black women had a 48% lower odds for receipt of influenza vaccination compared to White endometrial cancer survivors 8. In a longitudinal study of breast cancer survivors, Snyder et al. found that Blacks compared to Whites with breast cancer had a 55% lower odds of receiving an influenza vaccination 13. Possible explanations for these findings may relate to beliefs and recommendations. Cross‐sectional surveys among Medicare beneficiaries that have attempted to identify the reasons for racial differences in influenza vaccine status found that Blacks and Hispanics were more likely to have negative beliefs toward the influenza vaccine compared to Whites 16, 17, 19. Regardless of patient attitudes, provider recommendation of an influenza vaccination has been shown to positively influence vaccination rates 17, 19, 29. Conversely, a provider may not recommend a vaccine due to the lack of familiarity of the vaccine recommendations in the elderly 30. We were unable to measure these factors in the SEER‐Medicare dataset.

We found disparities in breast cancer screening with NH Black, and Hispanic endometrial cancer survivors having a lower odds for screening compared to White survivors. Prior studies have documented similar findings where Black endometrial cancer survivors more than 5 years after cancer diagnosis had a 26% lower odds of receipt of mammography 8, Black breast cancer survivors 1 year after diagnosis had a 54% lower odds of screening 13, and Hispanic women had a 26% lower odds for breast cancer screening compared to Whites 31. Geographic barriers to mammography screening locations and the perceived necessity for mammography may have contributed to disparities in breast cancer screening. Mobley et al. examined socio‐ecological predictors for mammography use among SEER‐Medicare beneficiaries in California, and found that women who lived in urban areas, segregated Hispanic neighborhoods, communities with high poverty rates, or areas where workers travel a long distance between work and home were less likely to utilize screening mammography 32. In this study, we found that a greater proportion of NH Black women and Hispanic women were classified as living in poverty, but we were unable to directly measure how poverty contributes to disparities in breast cancer screening. It is conceivable that poverty is a surrogate for other factors that are contributing to a lack of screening, such as reliable transportation to mammography centers and/or social support within in the community. Given that obesity is associated with the development of numerous cancers, including both endometrial cancer and breast cancer 33, it is concerning that many US women with early‐stage endometrial cancer are not being appropriately screened for breast cancer.

The proportion of women screened for diabetes mellitus within 5 years of endometrial cancer diagnosis was higher among Hispanic and Asian/PI women compared to NH White women. Prior studies have shown that among Asian and White Medicare beneficiaries, Asians were less likely to have had diabetes screening 34. A plausible reason for this difference is that we combined Pacific Islanders with Asians, and Pacific Islanders have a high prevalence of obesity and medical comorbidities 35. Furthermore, the higher prevalence of diabetes mellitus in minority populations 36 could have prompted providers to screen these women more than NH White women.

In the 5 years after endometrial cancer diagnosis, we found that over 90% of all women received screening tests for cholesterol, well above the Healthy People 2020 target of 82% for cholesterol screening during a 5‐year time span 28. These findings are consistent with other national reports that have shown that approximately 90% of women over age 65 receive cholesterol screening without regard to race 37.

There are limitations in using the SEER‐Medicare database to address the question of receipt of preventive services. First, there is the potential for measurement error. If any screening test was ordered with a diagnostic billing code, we would not be able to correctly identify that test as a screening test. For example, a mammogram could be converted to a diagnostic mammogram during the same visit if a lesion was detected during the exam 38. However, we expect this to be nondifferential misclassification by race, and it would bias any association with race toward the null. To minimize this bias, we only considered screening mammography ICD‐9 codes in this analysis. Furthermore, there is the possibility for residual confounding given that we were not able to account for individual level socioeconomic characteristics which could be associated with race/ethnicity as well as the utilization of preventive services. Lastly, the results may have limited external validity as we were only able to study women 66 years and older with Medicare of whom the majority are concentrated in urban areas. There may be differences in the utilization of preventive services for endometrial cancer survivors who are younger, have private insurance, or live in rural areas.

## Conclusions

Women with early‐stage endometrial cancer have a low likelihood of cancer‐related death 1, 3 and measures available to prevent other medical comorbidities are no less important in them than in other women. We found that in the US Medicare population, women with early‐stage endometrial cancer had a relatively low utilization of preventive measures recommended by the USPTSF and the CDC, namely influenza immunization and screening mammography. In addition, Black and Hispanic women had particularly low levels of receipt of influenza vaccination and breast cancer screening. The reasons behind these observed gaps are complex; likely influenced by practice patterns as well as economic, social, and behavioral factors. Future work should focus on understanding how these influences interact to create barriers that sustain inequities in care. Suggestions to improve compliance and reduce disparities in utilization of preventive services following a diagnosis of endometrial cancer include educating patients and providers about general health screening and screening that is based on comorbidities such as obesity. Current endometrial cancer surveillance guidelines recommend that cancer survivors have a history and physical exam every 3 to 6 months in the first 12 months after diagnosis and then every 6 to 12 months in the 24 to 60 months after diagnosis 23. Cancer survivorship plans can be developed to include preventive health care recommendations thereby promoting the utilization of preventive services in this population.

## Conflict of Interest

All authors have no financial disclosures or conflicts of interests to report.

## Supporting information


**Appendix S1.** Preventative health and vaccine recommendations for adults > = 65 years.
**Appendix S2.** Codes for screening tests or procedures up to 5 years after endometrial cancer diagnosis.
**Appendix S3.** Causal diagram of potential confounders.
