# Supplementary material for: Racial disparities in the utilization of preventive health services among older women with early‐stage endometrial cancer enrolled in Medicare
Source: Cancer Med. 2017 Aug 4;6(9):2153–63. doi: 10.1002/cam4.1141 (PMC5603841; doi:10.1002/cam4.1141)

| **Appendix 1: Preventative health and vaccine recommendations for adults >= 65 years** | | | |
| --- | --- | --- | --- |
| Screening/Prevention | Year implemented | Age | Method |
| Medicare well visit^1^ | 2011 | 65 years and older | Annually |
|  |  |  |  |
| Breast cancer^2^ | 2002 | 50-74 years | Biennial Mammography |
|  |  |  |  |
| Influenza Vaccine^4^ | 1993 | 65 and older | Once annually |
|  |  |  |  |
| Diabetes Mellitus type 2^2^ | 2008 | Asymptomatic adults with sustained BP 135/80 mmHg, | FPG, 2 hour post load, Hemoglobin A1C (ideal interval unknown: ADA recommendations Q3yrs). Note consider 10 year CHD risk and if DM screen will be helpful |
|  |  |  |  |
| Lipid disorder^2^ | 2008 | Women >=45 at increased risk for CHD | Optimal interval not known, 5 year interval reasonable, sooner if levels borderline |
| ^1^Centers for Medicare and Medicaid Services/Affordable Care Act | | |  |
| ^2^U.S. Preventive Services Task Force | |  |  |
| ^3^ American Association of Clinical Endocrinologists | | |  |
| ^4^ Centers for Disease Control and Prevention Advisory Committee on Immunization Practices | | | |

| **Appendix 2: Codes for Screening tests or procedures up to 5 years after endometrial cancer diagnosis** | | | | | |
| --- | --- | --- | --- | --- | --- |
| **Test/procedure** |  | **CPT* code** | **ICD-9~ Diagnosis code** | **ICD-9 Procedure code** | **HCPCS^ code** |
| **Influenza vaccine** |  |  |  |  |  |
|  |  | 90654-90664 | V04.81 | 99.52 | G0008 |
|  |  | 90666-90668 |  |  |  |
|  |  | 90672 |  |  |  |
|  |  | 90685-90686 |  |  |  |
|  |  | 90724 |  |  |  |
| **Lipid screen** |  | **CPT code** | **ICD-9 Diagnosis code** | **ICD-9 Procedure code** | **HCPCS code** |
|  |  | 80061 | V77.91 | 272.2 | G0054 |
|  |  | 82465 | V81.0 | 272.3 |  |
|  |  | 84478 | V81.1 |  |  |
|  |  | 83718 | V81.2 |  |  |
|  |  | 83719 |  |  |  |
|  |  | 83721 |  |  |  |
| **Mammography** |  | **CPT code** | **ICD-9 Diagnosis code** | **ICD-9 Procedure code** | **HCPCS code** |
|  |  | 76092 | V76.12 | 87.37 | G0202 |
|  |  | 77057 | V76.11 | 89.36 |  |
|  |  |  |  |  |  |
|  |  |  |  |  |  |
|  |  |  |  |  |  |
| **Diabetes Screening** |  | **CPT code** | **ICD-9 Diagnosis code** | **ICD-9 Procedure code** | **HCPCS code** |
|  |  | 82946 | V77.1 |  |  |
|  |  | 82947 |  |  |  |
|  |  | 82948 |  |  |  |
|  |  | 82950 |  |  |  |
|  |  | 82951 |  |  |  |
|  |  | 82952 |  |  |  |
|  |  | 82960 |  |  |  |
|  |  | 82962 |  |  |  |
|  |  | 83036 |  |  |  |
|  |  | 83021 |  |  |  |
| **Well visit** |  | **CPT code** | **ICD-9 Diagnosis code** | **ICD-9 Procedure code** | **HCPCS code** |
|  |  |  | V70.0 |  | G0402 |
|  |  |  |  |  | G0438 |
|  |  |  |  |  | G0439 |
| *Current Procedural Terminology | |  |  |  |  |
| ~International Classification of Diseases Ninth Edition | | |  |  |  |
| ^ Healthcare Common Procedure Coding System | | |  |  |  |

**Appendix 3: Causal diagram of potential confounders**


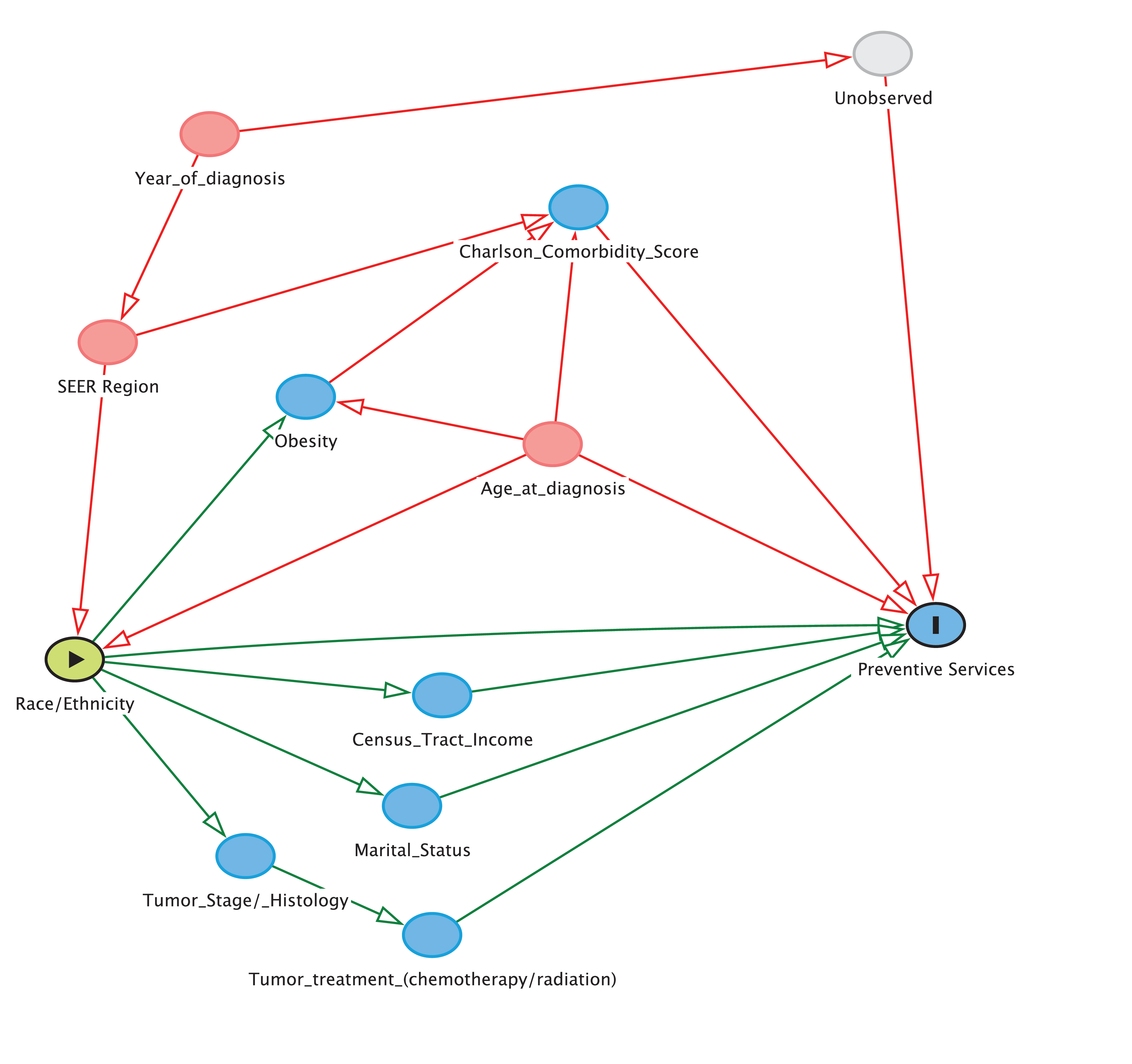

Supplement: Supplementary file 1 — Appendix S1. Preventative health and vaccine recommendations for adults > = 65 years. Appendix S2. Codes for screening tests or procedures up to 5 years after endometrial cancer diagnosis. Appendix S3. Causal diagram of potential confounders. [file CAM4-6-2153-s001.docx]
